# Cancer Patients and the COVID-19 Vaccines: Considerations and Challenges

**DOI:** 10.3390/cancers14225630

**Published:** 2022-11-16

**Authors:** Muna Almasri, Khalifa Bshesh, Wafa Khan, Malik Mushannen, Mohammad A. Salameh, Ameena Shafiq, Ahamed Lazim Vattoth, Nadine Elkassas, Dalia Zakaria

**Affiliations:** 1 Division of Medical Education, Weill Cornell Medicine-Qatar, Qatar Foundation, Education City, Doha 24144, Qatar; 2 Department of Obstetrics and Gynecology, Mayo Clinic, Rochester, MN 55902, USA; 3 Neurological Institute, University Hospitals Cleveland Medical Center, Cleveland, OH 44106, USA; 4 Peninsula Medical School, University of Plymouth, Plymouth PL4 8AA, UK; 5 Division of Premedical Education, Weill Cornell Medicine-Qatar, Qatar Foundation, Education City, Doha 24144, Qatar

**Keywords:** cancer, cancer therapies, COVID-19, vaccination, immunogenicity, safety, vaccine hesitancy

## Abstract

**Simple Summary:**

Coronavirus disease 2019 (COVID-19) is the greatest present-day public and global health challenge, and patients with cancer are especially vulnerable, emphasizing the importance of vaccination. However, little is known about the effects of cancer and treatment on vaccine effectiveness and its safety. The aim of this review is to explore current literature regarding the immune response rate and safety profile of COVID-19 vaccination in patients with solid and hematologic cancers and those receiving various forms of treatment. Immune response rates were described to be lower amongst cancer patients, especially those with hematologic cancers, and those receiving chemotherapy, radiotherapy, or immunosuppressants. Nevertheless, sufficient immune response was still generated in many patients, and vaccination was overall described to be safe and well-tolerated, therefore supporting vaccine encouragement.

**Abstract:**

Few guidelines exist for COVID-19 vaccination amongst cancer patients, fostering uncertainty regarding the immunogenicity, safety, and effects of cancer therapies on vaccination, which this review aims to address. A literature review was conducted to include the latest articles covering the immunogenicity and safety of COVID-19 vaccination in patients with solid and hematologic cancers receiving various treatments. Lower seropositivity following vaccination was associated with malignancy (compared to the general population), and hematologic malignancy (compared to solid cancers). Patients receiving active cancer therapy (unspecified), chemotherapy, radiotherapy, and immunosuppressants generally demonstrated lower seropositivity compared to healthy controls; though checkpoint inhibition, endocrine therapy, and cyclin dependent kinase inhibition did not appear to affect seropositivity. Vaccination appeared safe and well-tolerated in patients with current or past cancer and those undergoing treatment. Adverse events were comparable to the general population, but inflammatory lymphadenopathy following vaccination was commonly reported and may be mistaken for malignant etiology. Additionally, radiation recall phenomenon was sporadically reported in patients who had received radiotherapy. Overall, while seropositivity rates were decreased, cancer patients showed capacity to generate safe and effective immune responses to COVID-19 vaccination, thus vaccination should be encouraged and hesitancy should be addressed in this population.

## 1. Introduction

The novel coronavirus disease 2019 (COVID-19) has affected millions of lives around the world and has become the largest public and global health challenge of our time. Several studies and clinical observations in patient populations around the world have shown that individuals with advanced age and co-morbid conditions have a higher rate of morbidity and mortality from severe acute respiratory syndrome coronavirus 2 (SARS-CoV-2) infection.

### Cancer Patients and COVID-19

Patients with cancer have demonstrated higher infection rates from COVID-19, increased morbidity, more severe progression of disease, prolonged hospital stays, and increased risk of severe clinical events, as compared to those without cancer [1,2]. Furthermore, cancer patients may face increased contact with COVID-19 infected patients due to regular exposure to the hospital setting for anti-cancer treatment [2]. Combined, these factors place an urgent need for protection against COVID-19 amongst the cancer population. Worldwide, cancer societies have insisted that patients with cancer be considered a high-priority population for COVID-19 vaccination, despite the exclusion of patients with an active cancer status from clinical trials [3,4]. In particular, it has been suggested that patients with later-stage cancer are even more susceptible to SARS-CoV-2 [2]. Furthermore, cancer survivors have also demonstrated increased severity of COVID-19 symptoms, suggesting an incomplete recovery of immune surveillance methods and weakened defense system [2].

**Figure 1:**
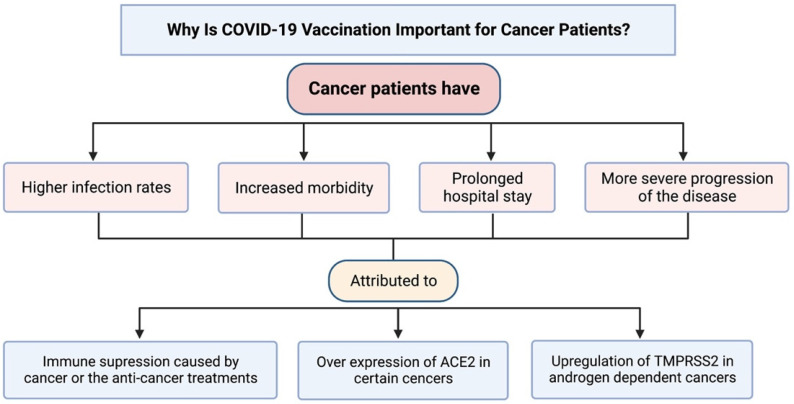
Reasons supporting the importance of COVID-19 vaccination in cancer patients, and mechanisms attributed to cancer patients’ increased susceptibility, severity, and morbidity from COVID-19 disease.

A few mechanisms have been hypothesized to explain why cancer patients are increasingly susceptible to higher infection rates and more severe disease. Patients with cancer, especially hematological cancers, commonly have a dysregulated immune system either caused by the cancer itself or the treatment they receive [5]. In cancer patients, the immune suppressive M2 macrophages are activated which inadvertently allows for tumor progression. This immunosuppression also disrupts the antiviral immune response, weakening the host’s defense against infections such as COVID-19. Additionally, the receptor that SARS-CoV-2 interacts with to infect host cells is the angiotensin-converting enzyme 2 (ACE2) receptor [6]. Studies have reported that ACE2 expression is increased in certain cancers, including lung cancer, which may contribute to the increased susceptibility [7]. Another link may involve the host transmembrane serine protease 2 (TMPRSS2), which is required by SARS-CoV-2 to release its viral RNA. As the protease is androgen-regulated, upregulation of the protease is seen in androgen-dependent cancers such as prostate cancer. One study showed that prostate cancer patients that were treated with androgen deprivation therapy (ADT) had significantly reduced COVID-19 infection rates as compared to patients without ADT [8]. This may indicate that upregulation of TMPRSS2 plays a role in the pathogenicity for certain cancers. Figure 1 summarizes the reasons behind the importance of vaccinating cancer patients against COVID-19, as well as the attributed mechanisms to this population’s vulnerability to the disease.

Though the extent of benefit from vaccination in this population is not fully delineated, accumulating data supports that COVID-19 vaccines are safe and demonstrate efficacy in cancer patients [9], which this review explores further. This narrative review aims to summarize the latest available information regarding the immunogenicity and safety of COVID-19 vaccines in cancer patients of different types (e.g., solid versus hematological) receiving different cancer treatments (e.g., chemotherapy, radiotherapy) in hopes of communicating an optimal strategy to better manage the health of this vulnerable population. The COVID-19 vaccines mentioned in this review span the mRNA vaccines (Pfizer, New York, NY, USA, Moderna, Cambridge, MA, USA), viral vectored vaccines (AstraZeneca, Cambridge, UK, Johnson & Johnson/Janssen, Beerse, Belgium), and inactivated vaccines (Sinopharm, Beijing, China).

## 2. Immunogenicity of the COVID-19 Vaccines in Solid and Hematologic Cancer Patients

In general, some studies reported lower immunogenicity in the cancer patients as compared to healthy controls. For example, Palaia et al., reported that there was no significant difference in the mean antibody titer one month after the second dose of Pfizer vaccination in 44 cancer patients as compared to the controls [10]. However, a significantly lower titer was reported three months after the second dose in cancer patients as compared to the healthy controls. Similarly, a study by Yasin et al., reported a significantly lower seropositivity rate in 661/776 (85.2%) of cancer patients compared to 697/715 (97.5%) of the control group after the second dose of the Sinovac vaccine [1]. Another study conducted by Ligumsky et al., reported that 287/326 (88%) of cancer patients had significantly lower immunoglobulin G (IgG) titer following the second dose of the Pfizer vaccine (median IgG titer 931 AU/mL) as compared to 159/164 (96.95%) of the healthy controls (median IgG of 2817 AU/mL) [9].

When comparing patients with solid malignancies to healthy controls, median neutralizing antibody titers were found to be similar between solid cancer patients and controls (*p* = 0.566), indicating comparable protection in seropositive people who mounted an immune response to vaccination [11]. On the other hand, a study conducted by Massarweh et al., found that patients with primary brain tumors had an 88.2% seroconversion rate following the Pfizer vaccine, though these patients demonstrated significantly lower IgG titers than controls (*p* = 0.002) [12].

Fendler and colleagues conducted a longitudinal prospective cohort study entitled *COVID-19 antiviral response in a pan-tumor immune monitoring study (CAPTURE)*. This study analyzed 585 patients, of which 76% had solid malignancies and 24% had hematological malignancies. Amongst these patients, 74% received two doses of the AstraZeneca vaccine, and 26% received two doses of the Pfizer vaccine [13]. The CAPTURE study found that most solid cancer patients developed durable humoral responses to SARS-CoV-2 infection or vaccination, with a seroconversion rate of 44% after the first dose and 85% after the second dose. Additionally, when the immunogenicity was compared in patients with solid or hematologic malignancy (HM), Fendler et al., described a reduced neutralizing antibody response in HM compared to solid cancer patients or controls [13]. Interestingly, Singer et al., reported that antibody titers against the SARS-CoV-2 spike (S) protein were significantly higher in solid cancer patients over HM indicating that the type of cancer may also affect the immunogenicity [14]. Furthermore, Agha et al., reported that 31/67 (46.3%) of patients with HM such as B-cell chronic lymphocytic leukemia (CLL), lymphomas, or multiple myeloma (MM) did not produce antibodies to COVID-19 messenger RNA (mRNA) vaccination [15]. In fact, patients with CLL were significantly less likely to respond to vaccination compared to other hematologic malignancies (23.1% vs. 61.1%; *p* = 0.01) [15]. Additionally, the United Kingdom’s prospective observational study evaluating COVID-19 vaccine responses in individuals with lymphoma (PROSECO) documented that 52% of patients with B-cell lymphoma undergoing active cancer treatment who received two vaccine doses had undetectable humoral response, though 70% of patients with indolent B-cell lymphoma showed increased antibody response after a booster dose [16]. Patients with hematological malignancies demonstrating a lower immune response as compared to solid tumors is, in part, because the disease process in hematological malignancies such as acute lymphoma, leukemia, and multiple myeloma is widespread and invasive and is particularly disruptive to the immune system [17]. In addition, treatments for hematological malignancies in general are more immunosuppressive, often leading to myelosuppression and lymphodepletion [17].

The above studies have shown evidence of lower seropositivity in cancer patients as compared to healthy controls and lower seropositivity in patients with hematologic malignancies as compared to those with solid cancers. However, in addition to the implication of the disease itself, cancer treatment affects the immunogenic response to vaccine which has been reported in numerous studies. The next sections summarize the findings of the studies that compared the seropositivity of cancer patients who received specific types of treatments with healthy controls or compared the seropositivity in those who received different types of treatments.

### 2.1. Lower Seropositivity in Patients Receiving Active Therapy Compared with Healthy Controls or Patients Not Receiving Active Therapy

Unlike the long-term anti-cancer treatments which are used to maintain remittance, active treatments are used to cure cancer, such as chemotherapy, radiotherapy, and others. This section reports the findings of the studies that mainly compared seropositivity in patients receiving active treatments with control groups, without specifying the individual effects of the different treatments. Nelli et al., reported that the median IgG titer in cancer patients without active treatment (control group) was more than twofold of that in cancer patients undergoing active treatment (exposed group) prior to receiving the second dose of either the Pfizer or Moderna vaccine. The median IgG titer 8 weeks after receiving the second dose in the control group had an approximately 15-fold increase compared to the exposed group [18]. A study conducted by Cavanna et al., showed that out of a total 257 cancer patients (85.2% on active treatments), 195 (75.88%) were seropositive 15–42 days after the second dose of the Pfizer or Moderna vaccine in comparison to 100% seropositivity in the healthy control group. Additionally, the median IgG titer in patients (118 AU/mL) was significantly lower than that in the control group (380.5 AU/mL) (*p* < 0.001) [19]. Furthermore, out of 195 seropositive cancer patients, 36 received no treatment, 84 received chemotherapy, 15 received immunotherapies, 26 received biological therapy, 24 received a combination of chemotherapy and biological therapy, and 10 received a combination of chemotherapy and immunotherapy [19]. Cavanna et al., reported in their second study that, following the second dose of either the Pfizer or the Moderna vaccine, 75/115 cancer patients (65.22%) were seropositive in comparison to 100% in the control group [20]. Furthermore, patients with HM yielded the lowest seroconversion rate (42.86% seroconverted vs. 70.21% of patients with solid tumor, *p* = 0.02). The differences in seroconversion were insignificant between patients who received active cancer therapy as compared to patients who did not (64.00% versus 73.33%). Furthermore, no significant difference was observed between the different individual treatments (chemotherapy 63.64%, immunotherapy 52.94%, biologic therapy 76.92%, hormone therapy 75.00%, and no treatment 73.33%) [20]. Overall, these studies support both decreased seropositivity rates as well as decreased antibody concentrations in patients receiving active cancer therapy compared to healthy controls, but no difference compared to cancer patients not receiving active therapy.

The next sections discuss the effect of specific types of treatments on the seropositivity of cancer patients after receiving different COVID-19 vaccines.

### 2.2. Lower Seropositivity in Cancer Patients Receiving Chemotherapy Compared to Healthy Controls or Patients Receiving Other Treatments

Chemotherapy consists of cytotoxic drugs that can disrupt the process of mitosis in cell division or cause deoxyribonucleic acid (DNA) damage. However, the susceptibility of cancer cells to these drugs can vary greatly. Such drugs induce stress or damage to the cells, enough to induce apoptosis [21]. Most therapeutic agents are delivered intravenously, but some are given orally, and both provide systemic therapy. Combination chemotherapy is a type of administration strategy which utilizes multiple different drugs to decrease the risk of developing resistance and allows for reducing the doses and consequently lowering toxicity [22]. However, these drugs lack the ability to distinguish between cancerous and healthy body cells, therefore resulting in adverse side effects due to damage of healthy, rapidly dividing cells in the bone marrow, hair follicles, and digestive tract [23].

Chemotherapy has been implicated in altering the immunogenic response to the vaccine. In fact, some studies present it as a main player in the reduced immunogenicity in cancer patients. Comparing chemotherapy to other treatment modalities produces useful conclusions but results can be variable. For example, Figueiredo et al., found that the effect of chemotherapy was less pronounced as compared to immunotherapy [24]. On the contrary, a study by Grinshpun et al., found that immunotherapy was associated with higher mean antibody levels compared to chemotherapy in 172 cancer patients who received two doses of the Pfizer vaccine (*p* = 0.0017) [25]. With regards to other treatment modalities, Ariamanesh et al., looked at 364 patients with cancer of which 131 were receiving chemotherapy. Chemotherapy treatment was associated with lower rates of seroconversion (83.5%) compared to radiotherapy or hormonal therapy (97%) [26]. Likewise, the study by Yasin et al., showed that the seropositivity rates were 78.6% in the active chemotherapy group, 85.7% in the immunotherapy group, 86.0% in the targeted therapies group, 87.1% in the hormone therapy group, and 91.1% in those receiving no active treatment where chemotherapy was found to be significantly associated with lower seropositivity (*p* < 0.001) [1]. The findings of Agbarya et al., also supported the impaired seropositivity in cancer patients who received chemotherapy. They looked at 140 patients with solid malignancies and 215 controls and found the odds ratio (OR) for negative serology in cancer patients to be 7.35 times compared to controls after adjusting for age and gender [27]. However, it was notable that negative serology was found only in chemotherapy treated patients and not in the other treatments, such as immunotherapy [27]. The case-control study conducted by Addeo et al., reported significantly lower antibody titers after two doses of the Pfizer or Moderna vaccine in patients receiving chemotherapy compared to those only under clinical surveillance [28]. Similar results were obtained by Funakoshi et al., who reported significantly lower median antibody titer for the 24 cancer patients receiving chemotherapy (0.161, 95% confidence interval (CI) [0.07–0.857]) after the second dose of the Pfizer vaccine as compared to 12 healthy controls (0.644, 95% CI [0.259–1.498]) (*p* < 0.0001) [29]. Likewise, Palaia et al., found that cancer patients who were receiving active chemotherapy treatment had lower median antibody titer one month and three months after the second dose as compared to those who were not receiving active chemotherapy [10]. Interestingly, the specific effect of the chemotherapy treatment and its extent varies depending on the drug at hand. A study by Ruggeri et al., looked at the rates of seroconversion with the Pfizer vaccine at the time of and just before the second dose, and then 8 weeks later [30]. It found that alkylating agents and tyrosine kinase (TK) inhibitors caused a significant reduction in IgG titers after the first dose of the vaccine and before the second dose. This effect was mitigated after the second dose. On the other hand, mammalian target of rapamycin (mTOR) inhibitors caused reductions in IgG titers after both doses at 8 weeks [30].

Overall, the above-mentioned studies consistently demonstrated significantly lower seropositivity and antibody titers amongst cancer patients receiving chemotherapy, including alkylating agents, TK inhibitors, and mTOR inhibitors, compared to healthy controls.

### 2.3. Effect of Radiotherapy on the Immunogenicity of COVID-19 Vaccines in Cancer Patients

Radiotherapy aims to treat cancer locally by using ionizing radiation, decreasing the chances of undesired damage in healthy body tissues, and thus reducing side effects. Despite local treatment, dosage of radiation must be limited as some nearby healthy cells are also destroyed during the process, resulting in adverse side effects such as fatigue and nausea [23].

Radiotherapy has also been associated with decreased seropositivity following vaccination. A subset of the Cancer, COVID-19 and Vaccination (CANVAX) prospective cohort study included 33 patients who had received thoracic radiotherapy, the majority of which had non-small cell lung cancer (NSCLC) or small cell lung cancer (SCLC) [31]. Of these patients, 14 had received stereotactic body, palliative, or definitive radiotherapy, 13 had received chemotherapy, and 9 had potentially immunosuppressive medical conditions; 79% of patients received radiotherapy prior to vaccination with either the Moderna, Pfizer, or AstraZeneca vaccine. This analysis found that antibody concentrations against the spike protein were significantly lower in the 33 patients treated with thoracic radiotherapy compared to vaccinated healthy controls (*p* = 0.01) [31]. However, though lower antibody concentrations were noted between patients with thoracic malignancies who received radiotherapy versus those who did not, the difference was not statistically significant (*p* = 0.07) [31]. Moreover, amongst patients receiving radiotherapy, those with immunosuppressive conditions (including those receiving chemotherapy) were found to have significantly lower antibody concentrations; 44% of patients with immunosuppressive conditions had antibody levels < 100 U/mL, compared to 13% without (*p* = 0.04) [31]. Overall, this study supported the notion that cancer patients receiving radiotherapy demonstrate significantly lower seropositivity compared to healthy controls, but an insignificant difference compared to patients with malignancy not treated with radiotherapy.

### 2.4. Effect of Checkpoint Inhibitors on the Immunogenicity of COVID-19 Vaccines in Cancer Patients

Immunotherapy involves stimulating the immune system to fight cancer cells. There are several types of immunotherapies, including the checkpoint inhibitor therapy (CPI). The immune system checkpoints are vital for regulation, and some reduce the action of T-cells. CPI therapy consists of drugs which block these checkpoints and allow T-cells to continue attacking the cancer cells [32]. Current drugs target molecules such as programmed cell death protein 1 (PD-1), and programmed death ligand-1 (PD-L1) which play a role in T-cell regulation [33,34].

Immunotherapies with immune amplifying effects have been associated with an adjusted response to the vaccine. Figueiredo and others found a significant drop in the four-to-six month antibody level of those with solid tumors receiving immune CPI therapy compared to those receiving other treatment modalities, including chemotherapy (*p* = 0.004) [24]. Notably, the reduction in antibody levels was more pronounced in those who received the vaccine after starting the CPI therapy compared to those who received it before CPI therapy [24]. On the contrary, the study by Naranbhai found that those receiving immune checkpoint (ICP) modulators tended to have a higher neutralization [35]. Ma et al., recruited 545 cancer patients who received either progression cell death-1 blockers (PD-1B), COVID-19 vaccination, or both in three matched cohorts and compared them with a non-cancer control group of 206 participants. Seropositivity was detected in 68.1%, 71.3%, and 80.5% of the vaccinated cancer patients who received PD-1B, who did not receive PD-1B, or the healthy control subjects respectively [36]. The study concluded that patients with cancer tolerated the COVID-19 vaccines well and that the PD-1B treatment did not affect the seroconversion rate following vaccination. However, the seroconversion rate was generally lower in the cancer patients as compared to the healthy control participants [36]. Similarly, Funakoshi et al., reported that the median antibody titer for the 17 cancer patients receiving ICP treatment was significantly lower after the second dose of the Pfizer vaccine (0.241, 95% CI [0.063–1.205]) as compared the 12 healthy controls (to 0.644, 95% CI [0.259–1.498]) (*p* = 0.0024) [29]. Another study by Lasagna et al., looked at 88 patients treated with PD-L1 inhibitors and assessed their response to the Pfizer vaccine at three weeks. It found that recipients of this treatment were able to illicit a robust T-cell (CD4 and CD8) response to the vaccine, demonstrating the vaccine’s ability to include both types of adaptive responses [37]. A continuation study of the same cohort found significantly waning immunity at 6 months, particularly for those who were SARS-CoV-2 naïve [38]. These studies indicate that while seroconversion rate was generally unaffected, the antibody titers were significantly decreased in patients receiving immunomodulating therapy, though adaptive T-cell responses amongst this patient population remained intact.

### 2.5. Lower Seropositivity in Cancer Patients Receiving Immunosuppressives Compared to Healthy Controls or Patients Receiving Other Treatments

Immunosuppressive drugs are given to reduce the activity of the immune system [39]. It is also important to note that immunosuppression can also be a result of cytotoxic drugs used in chemotherapy, as they mainly target cells that rapidly divide, including T-cells [40]. Deliberate immunosuppression is vital for preventing body rejection after organ transplants, which is common amongst cancer patients. These drugs are also given when treating graft-versus-host disease (GvHD) following bone marrow transplants [41]. Certain types of immunotherapies may lead to immunosuppression such as the anti-CD20 treatments which block the B cells and the chimeric antigen receptor (CAR) T-cells. The CAR T-cell method is the genetic modification of T-cell receptor proteins which allows for antigen binding and T-cell activation via a single receptor. For cancer therapy, these T-cells can be engineered to recognize specific antigens found on tumor cells only. This is done by the extraction of the T-cells from patients, genetic modification, then reintroduction into the body. The CAR T-cells then become cytotoxic once they attach to the tumor cells [42]. Derivation of T-cells can be from either the patient’s blood or a healthy donor [43].

Several studies demonstrated reduced immunogenicity in response to the vaccine in the setting of immunomodulating therapy. One frequently reported treatment is anti-CD20, which blocks a B-cell surface protein involved in B-cell activation and hence alters immune response. Addeo and others looked at 131 cancer patients, four of which received anti-CD20 treatment and found that none of the latter developed a humoral response to the vaccine [28]. Another study conducted by Thakkar et al., looked at 200 cancer patients with solid or hematologic cancers in New York City and found that those with HM had an 85% rate of seroconversion after receiving two doses of the Pfizer or Moderna vaccine or one dose of the Johnson & Johnson/Janssen adenoviral vaccine. This rate was reduced to 70% if the patients were receiving anti-CD20 therapy (*p* = 0.0001) [44]. Other immunosuppressive therapies that were associated with reduced immunity include stem cell transplantation with seroconversion rate of 73% (*p* = 0.0002), and CAR-T cell therapy with zero of the three patients who received it seroconverting (*p* = 0.0002) [44]. Further, a paper by Shapiro and colleagues examined the efficacy of the Pfizer or Moderna booster dose in 88 cancer patients. This study noted significantly lower seroconversion rates amongst patients receiving anti-CD20 therapy in the last 3.9 months. Of the 88 patients, 32 were seronegative prior to the booster, and fourteen (44%) remained seronegative following the booster, including two patients undergoing CAR T-cell therapy and two receiving stem cell transplants; 18/32 seroconverted following the booster (*p* = 0.000062) [45]. Similar results were obtained by Peeters et al., who conducted a multicohort study, whose results revealed extremely low antibody response in HM patients receiving rituximab [46]. Furthermore, the PROSECO study revealed that 60% of fully vaccinated patients demonstrated undetectable antibodies within 12 months of receiving anti-CD20 therapy [16].

Regarding corticosteroid therapy, a study by Nelli et al., found that amongst 311 patients who received two doses of the Pfizer vaccine, receiving initial corticosteroid therapy was associated with reduced IgG response (*p* = 0.005) [47]. Similarly, Naranbhai et al., looked at the response of 656 patients who received two doses of Pfizer or Moderna, or one dose of the Johnson & Johnson/Janssen vaccine and found that patients currently on steroid therapy had lower antibody titers (*p* = 0.003) [35].

Daratumumab, a CD38 inhibitor, was associated with a similar decrease in immunogenicity. By targeting CD38 on plasma cells, daratumumab thus depletes antibody production and reduces vaccine immunogenicity, potentially explaining this finding. In patients with multiple myeloma, active treatment with proteasome inhibitor-based or Imids-based had a higher likelihood of response to Pfizer vaccine than treatment which included daratumumab (92.9% vs. 50%; *p* = 0.003) [48].

In summary, various modalities of immunosuppressive cancer treatment, including anti-CD20 agents, corticosteroids, and CD38 inhibitors, are associated with significantly lower seroconversion rates as well as immunogenicity, likely due to the mechanism of action of immunosuppressives.

### 2.6. Effect of Other Treatments on the Immunogenicity of COVID-19 Vaccines in Cancer Patients

#### 2.6.1. Endocrine Therapy

The main function of hormonal therapies is to block or change certain hormone systems to slow down the growth of specific cancers. For example, hormonal therapy is used to treat estrogen receptor positive breast cancer [49].

Referring to the study conducted by Addeo and colleagues, of 131 patients, 15% received endocrine therapy prior to vaccination with Pfizer or Moderna. Some 94% of patients receiving endocrine therapy were seropositive after dose one, and 100% demonstrated seropositivity after dose two, with excellent median antibody titers (>2500 U/mL). Overall, it was found that endocrine therapy had no discernable impact on seropositivity at a minimum of three weeks post-vaccination series, with similar outcomes to patients receiving no therapy [28].

#### 2.6.2. Cyclin Dependent Kinase Inhibitors

A prospective study conducted by Cortés et al., found that amongst 26 patients being treated with cyclin dependent kinase inhibitors (CDKi), rates of humoral response in patients treated with CDK4/6i were similar to healthcare worker (HCW) controls [50]. Some 100% of the CDK4/6i cohort showed positive serology after the first and second doses, with no significant difference in anti-S IgG levels. However, there was a significantly lower cellular response in CDK4/6i recipients compared to HCW; the anti-S CD4 response was found to be 59.7% and 91.7% amongst CDK4/6i and HCW cohorts, respectively (*p* = 0.001), and the anti-S CD8 response was 55.6% and 94.4% amongst CDK4/6i and HCW cohorts, respectively (*p* < 0.001) [50]. There was no known predictive factor for poor cellular response amongst patient characteristics. Though seropositivity and humoral response were seemingly unaffected by CDK4/6i, the difference in cellular response was thought to be due to CDK4/6i induced neutropenia via reversible bone marrow suppression by cell cycle arrest [50].

#### 2.6.3. Stem Cell Transplants

Khan and colleagues conducted a prospective, observational longitudinal cross-sectional study of 453 cancer patients undergoing treatment or who received stem cell transplantation (SCT). Within this population, 114 patients received SCT [51]. Patients receiving allogeneic SCT, autologous SCT, or CAR T-cell therapy demonstrated adequate levels of anti-S titers (>100 U/mL) at one and three months following the second dose of either the Moderna, Pfizer, or Johnson & Johnson/Janssen vaccine; the geometric mean titer amongst the SCT group was 325.35 (95% CI [149.93–706.01]) after one month, and increased to 454.36 (95% CI [237.48–869.32]) after three months [51]. Anti-S titers > 100 U/mL or higher are associated with protection and thus higher vaccine effectiveness [51].

#### 2.6.4. Combination Treatment with Chemotherapy and Immunotherapy

Combining treatments was reported to intensify the anti-cancer effect compared to receiving individual therapies. A study by Massarweh et al., looked at 102 patients with cancer and their seroconversion in response to two doses of the Pfizer vaccine. Multivariate analysis of the cohort found that the only variable significantly associated with lower IgG values was combination treatment with both chemotherapy and immunotherapy, compared to chemotherapy or immunotherapy alone (*p* = 0.001) [52]. However, though Grinshpun et al., found the seroconversion rate amongst patients receiving immunotherapy plus chemotherapy to be lower (10/12, 83.3%) than those receiving immunotherapy alone (32/34, 94.1%), this difference was not significant [25].

### 2.7. Effect of the Number of the COVID-19 Vaccine Doses on the Seropositivity of the Cancer Patients

The study by Monin et al., followed individuals who received the Pfizer vaccine and showed that a single dose of 30 μg failed to induce seroconversion in most patients with cancer. However, the same dose induced T-cell responses in a majority of healthy controls and solid cancer patients even though many of them were seronegative [4]. This should support prioritizing the cancer patients to receive an early (day 21) second dose of the Pfizer vaccine. Other studies reported that cancer patients were seropositive after receiving the second dose of different types of vaccines, including those who were on active cancer treatments. For example, Goshen-Lago et al., demonstrated seropositivity in 25/86 cancer patients (29%) with a median titer of 42.3 compared with 220/261 (84%) in controls (*p* < 0.001), who had a median titer of 72.0 following the first dose. However, the rates increased to 187/218 patients (86%) following the second dose of the Pfizer vaccine. Of the 187 seropositive cancer patients following the second dose, 55% received chemotherapy, 38% received immunotherapy and 37% received biological agents [53]. Similarly, following the second dose of inactivated Sinopharm vaccine, 102/119 (85.7%) cancer patients were SARS-CoV-2 anti-spike IgG positive, 65 of which received endocrine therapy, trastuzumab, 18 received chemotherapy and 19 received radiotherapy. Furthermore, 104/119 (87.4%) were SARS-CoV2 anti-receptor binding domain (RBD) IgG (neutralizing antibody) positive, 66 of which received endocrine therapy, trastuzumab, 19 received chemotherapy and 19 received radiotherapy [54]. Additionally, despite postulated lower immunogenicity amongst patients with solid malignancy, antibody titers were found to increase following the second dose of the COVID-19 vaccine, highlighting the need for a third. For example, Trontzas et al., found that in patients with thoracic cancer (93.1% lung cancer, including NSCLC, SCLC, and pleural malignant mesothelioma), a second dose of the Pfizer, Moderna, or AstraZeneca vaccine given in patients with thoracic cancer increased antibody response [55]. Similarly, the serologic response rate amongst cancer patients increased from 14.2% after the first dose to 86% after the booster dose. Following the booster dose, 73.8% of the non-responders were receiving active chemotherapy and 40.5% were reported to receive targeted therapy [56]. When compared to controls, the serological response rate for cancer patients was lower at different time points [56].

## 3. Safety of the COVID-19 Vaccines on the Cancer Patients

Several new trials have reported that COVID-19 vaccines have shown similar safety profiles in cancer patients as compared to the general population. The most common local and systemic side effects were pain at injection site, myalgia, and fatigue [17]. These were mostly mild to moderate in severity in the general population and cancer patients [17]. The following sections describe the reported side effects post-COVID-19 vaccination in cancer patients.

### 3.1. Safety of the COVID-19 Vaccines in Cancer Patients with Different Treatments

Shulman et al., reported 1753 individuals who had received both doses of the Pfizer vaccine, out of which 570 had no cancer, 1183 had a history of cancer, and 211 were on active treatment. Treatment methods included surgery, radiation, chemotherapy, immunotherapy, hormone therapy, and targeted therapy. Rates of adverse events (AE) following vaccination were similar in both patients with and without cancer (73.3% vs. 72.5%; *p* = 0.71) [57]. The most common adverse event was local pain at the injection site, but these rates did not differ between patient category nor dose number. Patients with cancer and receiving therapy were significantly less likely to report pain at the injection following the first dose compared to patients with cancer not receiving therapy (30% vs. 41.4%; *p* = 0.002) [57]. Muscle pain after the first dose was significantly more common in patients with cancer compared to those without (16.5% vs. 11.9%; *p* = 0.012), but they had it for significantly shorter duration (mean 2.2 vs. 3.0 days; *p* = 0.04) [57]. The onset of symptoms was similar for both groups of cancer patients [57]. Another study conducted by Kian et al., revealed no significant difference between treatment protocol and development on the side effects of the Pfizer vaccine on cancer patients who received different types of anti-cancer treatments including chemotherapy, radiotherapy, immunotherapy, biological therapy, hormonal therapy, or who had a combination of two therapies. The overall incidence of side effects following either dose was 31%, similar to that reported in the safety data from the phase III trial (27%) [58].

In patients with urologic cancers, Kawaguchi et al., reported 214 patients, of which 180 received the AstraZeneca vaccine (2 patients received one dose, 178 received two doses). The patients were on different treatments where 36 patients received ICP inhibitors, 17 received systemic chemotherapy, 24 received molecular targeted therapy, 140 received hormonal therapy, and 6 patients received intravesical infusion therapy. Furthermore, bone modifying agents (BMA) were used in 28 patients, denosumab in 18, and zoledronic acid in 10 patients. Of the 180 vaccinated patients, 69 (38.3%) reported adverse events [59]. The study found that in their population, the incidence of adverse events was significantly higher in females than males (72.7% vs. 36.1%; *p* = 0.015) [59]. Of these, only one patient had to postpone therapy due to adverse reaction of immune checkpoint inhibitors, and only one due to adverse effects of the vaccine. Overall, the vaccine was found to be safe in urologic cancer patients receiving different types of therapy [59].

Furthermore, of 373 cancer patients at a London oncology center, 281 (75.4%) received mRNA (Pfizer or Moderna) vaccines, 88 (23.6%) received the adenoviral (Johnson & Johnson/Janssen) vaccine, and 4 (1.1%) received an unknown vaccine. Only four had received the second dose and three of them experienced new adverse events, including worsening pre-existing grade 1 pruritus, grade 2 transaminitis, and grade 2 hypercortisolism, all of which were not seen in other groups. These patients were on different types of anti-cancer treatments where 23.6% received hormonal therapy, 36.2% received parenteral chemotherapy and 15.3% received immunotherapy. It was found that patients receiving immunotherapy within 6 months of vaccination appear to be at lower risk of developing adverse events (OR 0.495, 95% CI [0.256–0.958]; *p* = 0.0037) [60]. Other negative independent predictors for developing vaccine-related systemic adverse events include: male gender (OR 0.632, 95% CI [0.400–0.999]; *p* = 0.049), presence of metastatic cancer (OR 0.548, 95% CI [0.347–0.867]; *p* = 0.010), receiving chemotherapy within 28 days of vaccination (OR 0.373, 95% CI [0.221–0.629]; *p* < 0.001) or receiving the Pfizer vaccine (OR 0.452, 95% CI [0.274–0.747]; *p* = 0.002) [60].

### 3.2. Safety of the COVID-19 Vaccines in Cancer Patients Receiving Radiotherapy

In patients receiving radiotherapy, Soyfer et al., reported two patients who developed acute skin reactions in previously irradiated areas after receiving the second dose of Pfizer vaccine. Both reactions were diagnosed as radiation recall phenomenon (RRP), which is an uncommon inflammatory skin reaction in areas previously receiving radiation therapy (RT). One case was treated with topical steroids and painkillers until resolved, and the other required no intervention and self-resolved [61]. Similarly, Marples et al., reported three cases of breast cancer female patients (62, 69 and 56 years old, respectively) who received radiotherapy and developed AstraZeneca vaccine induced RRP. The first case had left breast cancer and underwent bilateral mastectomy with reconstruction. Three days following her first dose of the AstraZeneca vaccine, she began having swelling, erythema, and pain in her left breast. She had no fever or systemic symptoms. She received a four-week course of steroids and received the second dose afterward without issues. The second case received adjuvant radiotherapy following left breast cancer treated with lumpectomy and sentinel node biopsy. She had fever, muscle aches, and lethargy three days after receiving the AstraZeneca vaccine, followed by pain and erythema in the left breast with left axillary pain. She was treated conservatively after ruling out collection or other serious issues, diagnosed with RRP, and never received the second dose. The third case had therapeutic reduction mammoplasty and sentinel node biopsy for multifocal lobular carcinoma of the right breast followed by whole breast radiotherapy. She had flu-like symptoms after receiving the AstraZeneca vaccine, and three days later experienced a warm, pruritic, and heavy sensation in her right breast, in a similar area to her radiation therapy. She received two days of antibiotics from her general practitioner, ruled out collection with ultrasound, and was diagnosed with RRP by her breast surgeon [62].

Scoccianti and colleagues evaluated overall tolerance to the Moderna vaccine with a cohort study involving 153 patients who had received either postoperative, definitive, palliative, or stereotactic ablative radiotherapy. Of this cohort, 33% of patients had no adverse events after the first dose, 38% had no AE after the second dose, and 20% had no AE after the first and second doses. It was concluded that overall, tolerance was not worse in radiotherapy patients compared to controls [63].

### 3.3. Safety of the COVID-19 Vaccines in Cancer Patients Receiving Immune Checkpoint Inhibitors

According to Waissengrin et al., of a total of 170 patients on immune checkpoint inhibitors, 134 patients received two vaccine doses, three patients received only one dose, and 33 did not receive the Pfizer vaccine at all. The most common side-effect after the first dose was localized pain at the site of injection (21%). Systemic side-effects included fatigue in five (4%), headache in three (2%), muscle pain in three (2%), and chills in one (1%) patient. More local and systemic side effects occurred following the second dose. The local adverse events include pain at injection site in 85 (63%), local rash in three (2%), and local swelling in twelve (9%) patients. The systemic side effects included muscle pain in 46 (34%), fatigue in 45 (34%), headache in 22 (16%), fever in 14 (10%), chills in 14 (10%), GI complications in 14 (10%), and flu-like symptoms in 3 (2.2%) patients [64]. None of the reported side effects required hospitalization or special intervention, and no immune-related AE were observed. Of note, patients were all matched by sex and birth year to compare the side effects; there was only a significantly higher rate of muscle pain in the immune checkpoint group compared to healthy controls following the second dose of the vaccine (*p* = 0.024) [64].

Strobel et al., reported 89 patients receiving immune checkpoint inhibitors. Four patients received one vaccine dose (one Johnson & Johnson/Janssen, 1 AstraZeneca, and 2 Pfizer) while 85 patients received two doses of an mRNA vaccine (76 Pfizer, 2 Moderna), AstraZeneca (8 patients) and mixed AstraZeneca and Pfizer (2 patients). Overall, they found that the rate of general side effects was lower than that seen in preliminary data from the vaccination studies [65].

Mei et al., compared 1518 vaccinated cancer patients (288 with one dose, 1134 with two doses, 96 with three doses) receiving camrelizumab alone or in conjunction with other therapies to unvaccinated patients. Compared with matched unvaccinated patients, a statistically greater percentage of vaccinated patients had mild AE ≤ 2 (33.8% vs. 19.8%; *p* < 0.001) following camrelizumab treatment [66].

One case report published by Au et al., described a 58-year-old male on anti-PD-1 monotherapy for metastatic colorectal cancer complicated by neurological and endocrine immune-related adverse events which required stopping and restarting the immunotherapy treatment. He finished his course of ICP therapy and received the first dose of the Pfizer vaccine 27 days later. Five days later, he presented with myalgia, diarrhea, fever, as well as elevated inflammatory markers, LDH, and thrombocytopenia. He was started on methylprednisolone for suspected cytokine release syndrome (CRS), and his symptoms resolved within seven days of treatment and was successfully restarted on the anti-PD-1 treatment. The vaccine was more likely to be the cause of CRS because the median time to CRS following immune checkpoint therapy is four weeks from initiation, and this patient began treatment 22 months prior [67].

Overall, the rates of AE varied across studies in cancer and non-cancer populations, however, reassuringly, reported AE tended to be mild, despite one isolated case of cytokine release syndrome following vaccination.

### 3.4. Lymphadenopathy Post-COVID-19 Vaccination in Cancer Patients

Lymphadenopathy signifies any inconsistency or abnormality in the lymph nodes; this abnormality can refer to the size, firmness, or number of lymph nodes in a given area of the body [68]. These lymphadenopathies can be a consequence of infections, including bacterial, viral, and parasitic causes. Recent studies have shown that unilateral lymphadenopathy has a great association with vaccines, such as the influenza vaccine, HPV vaccine, and BCG vaccine [69]. Post-vaccination lymphadenopathy may be falsely attributed to an oncological process in individuals who have been diagnosed with cancer, in remission, or at an increased risk of developing malignancies. As such, the possibility of post-vaccination lymphadenopathy must be considered in individuals who receive COVID-19 vaccinations, especially in patients with underlying or increased risk of oncological disorders [70]. Hypermetabolic lymphadenopathy refers to an abnormal lymph node which has an increased rate of metabolism, and this process can be visualized using an F-18 fluorodeoxyglucose positron emission tomography-computed tomography (FDG-PET-CT) scan. This scan utilizes radiotracers to map lesions that are metabolically active throughout the body, and FDG-PET-CT of the entire body is a component of the examination of cancer patients to evaluate progression of disease. However, FDG uptake is not unique to oncological disorders and can be seen in inflammatory or infectious conditions, which might be a consequence of vaccination [71].

Bshesh et al., reported 6022 cases of lymphadenopathy amongst COVID-19 vaccination recipients, of which 693 had confirmed malignancies [68]. All subjects in the studies conducted by Cohen et al., Eifer et al., and Bernstine et al., had underlying oncological disorders and were assessed for lymphadenopathy using FDG-PET-CT or other PET-CT tracers; relatively high rates of FDG-PET-CT positivity was reported amongst the cohorts [71,72,73]. Several studies have revealed that cancer patients had FDG-PET-CT hypermetabolic axillary lymph nodes and a focal hypermetabolic region in the ipsilateral deltoid muscle after Pfizer vaccination [71,73].

Cohen et al., reported that it may be difficult to distinguish between benign and malignant hyperactivity in lymph nodes, especially when vaccination was conducted on the same side, as the tumor is expected to undergo nodal drainage. Hence, the recommendation was made that patients with breast cancer, axillary lymphoma, and malignancy of the upper limb should not undergo vaccination in the arm that has a lymph node with expected nodal drainage of a tumor [72]. Placke et al., identified a further 8 patients with underlying melanoma or Meckel cell carcinoma who were misdiagnosed with lymph node metastases and underwent lymph node excision after COVID-19 vaccination [74]. The studies reiterate the notion that physicians must be aware of the possibility of post-vaccination lymphadenopathy when making diagnoses and management plans in patients with oncological disorders or complaints of a newly arising lymph node abnormality [75]. Studies reported that lymphadenopathy after COVID-19 vaccination should be considered reactive at first glance, due to stimulation of the immune system. If the patient has pre-existing unilateral cancer, vaccination should be given on the contralateral arm whenever feasible. Lymph nodes that are persistently enlarged several weeks later can be investigated for an underlying malignancy using fast, cost-effective methods such as fine needle aspiration [76]. In the context of breast cancer surgery, studies have recommended scheduling COVID-19 vaccination at least a week prior to surgery so that symptoms such as fever can be accurately attributed to a vaccination side effect rather than from surgery [77]. Moreover, vaccination is recommended at the contralateral side to the affected breast or the anterolateral thigh. Vaccination could also be done once the patient is recovered, one to two weeks after surgery. Ultimately, COVID-19 vaccination- induced reactive lymphadenopathy could possibly mask side effects of breast cancer surgery and the timing of vaccination should be modified accordingly [77]. If FDG-PET-CT is required urgently for cancer disease staging or treatment initiation, it is recommended that it should be attempted to be done prior to vaccination if possible. If the indication for FDG is not urgent, it is advised to delay or reschedule the scan. From clinical experiences from routine vaccinations, vaccine-induced lymphadenopathy typically arises within seven days of vaccination and resolves in twelve to fourteen days [78,79]. However, there have been reported instances of COVID-19 vaccine-related nodal FDG uptake up to four to six weeks after vaccination. Therefore, it is suggested that in patients with a cancer that is expected to be difficult to interpret with FDG after vaccination, the FDG-PET-CT scans should be delayed for at least two weeks unless there is a clinical indication which requires oncological imaging to be done sooner. More ideally, if oncological imaging is not urgent whatsoever, the FDG-PET-CT should be delayed for four to six weeks to circumvent possible confounding findings [80]. Becker et al., provides a set of recommendations for radiological management of post-vaccination adenopathy. They recommend observing for at least six weeks for resolution before referring for diagnostic imaging evaluation or biopsy of the nodes [81]. Moreover, if the cause of adenopathy is overwhelmingly likely to be due to recent vaccination than an underlying neoplasm, an expectant management strategy without default follow-up imaging is suggested. Imaging follow-up with ultrasound to assure resolution of adenopathy is recommended in high-risk patients such as one with ipsilateral breast cancer [81].

Lehman et al., recommends that vaccinations should not be delayed, and that vaccination history should be provided to the radiologist when they are interpreting imaging findings. In the context of known recent COVID-19 vaccination, the authors suggest that ipsilateral axillary lymphadenopathy can be managed clinically as there is a low pretest probability of malignant lymphadenopathy [82]. Lane et al., also suggests a conservative approach, but also recognizes that false nodal biopsy might be inevitable in certain breast cancer patients. To reduce false positive nodal findings post-vaccination, the history of COVID-19 vaccinations, number of doses and dates, as well as site and side of injection should be strictly documented at time of vaccine administration [83].

Locklin and Woodard state that mammographic findings such as trabecular and skin thickening, as well as increased echogenicity on ultrasound, can be visualized with edema secondary to poor lymphatic drainage or capillary leak, and should be kept in consideration as a potential etiology for breast edema after recent COVID-19 vaccination. They suggest that much like evaluations for suspected mastitis, imaging should be conducted for a short period to ensure resolution for patients with ipsilateral vaccinations histories. Inflammatory breast cancer can closely resemble inflammation and infection, and careful observation of resolution is important to not neglect cancer [84]. Chung et al., states that cortical thickness and its morphology on ultrasound are most helpful in distinguishing between malignancy or a benign reactive post-vaccination lymph node in the context of breast cancer. A cortical thickness threshold of 5.4 mm showed greatest specificity and accuracy for differentiating between benign and malignant processes. Moreover, completely hypoechoic nodes with no visible hila were observed in only malignant nodes [85]. Adin et al., reports a case of a patient with bilateral lymphadenopathy, one due to an ipsilateral breast malignancy and the contralateral one due to recent COVID-19 vaccination. The malignant node demonstrated asymmetric cortical thickening and marked cortical enhancement compared to the reactive node, further supporting Chung et al.’s findings [86]. Granata et al., identified that lymphangitis is also a possible consequence of COVID-19 vaccination. This must be considered with possible lymphadenopathies, to avoid alarmism in patients and physicians alike. Having knowledge of such a possibility allows for avoidance of economic waste via the utilization of several radiological studies in the hunt for a tumor that is likely not there [87].

## 4. Hesitancy/Acceptance of the COVID-19 Vaccination among Cancer Patients

The lower immunogenicity in cancer patients has been responsible for higher rates of infection in patients with cancer as well as higher risk of developing serious complication and death. The inadequate serological response can be attributed to malignancy-related immune dysregulation as well as a greater likelihood of co-morbid conditions in patients with cancer. In addition, cancer treatments contribute to immune suppression making it difficult to confer adequate immunity against many infections including COVID-19. As a result, patients with cancer are considered high risk and have been given priority in most vaccine rollout programs. However, hesitancy of receiving the COVID-19 vaccines among cancer patients has been widely reported.

### 4.1. Major Reasons for COVID-19 Vaccination Hesitancy

Studies from several countries explored the rate of vaccine acceptance among cancer patients and the reasons behind their hesitancy. With the initial rollout of vaccines, hesitancy among cancer patient was higher than the public. Similarly, in a cross-sectional study of 111 cancer patients from a Lebanese institution, 14.4% refused to get the vaccine while 30.6% were hesitant [88]. The main reason for refusal was the patient’s belief that vaccines were incompatible with their disease or treatment while hesitant patients wanted more information about the risk of vaccination in cancer patients and its efficacy [88]. Cross-sectional surveys done in Poland, Mexico, Bosnia and Herzegovina, Hong Kong, and Germany all revealed considerable rates of vaccine hesitancy in cancer patients, with vaccine acceptance rates of 58.8% out of 644 patients, 66% of 540 breast cancer patients, 41.8% of 364 cancer patients, 17.9% of 660 cancer patients and 62% of 101 patients respectively [89,90,91,92,93]. The studies reported the reasons for hesitancy/rejection, some of which were similar to the reasons for vaccination hesitancy among the public. For example, some patients were skeptical of how rapidly the vaccines were developed, with major concerns of the side effects post-vaccination. Others believed that their natural immunity could provide enough protection against COVID-19. However, some concerns were unique to cancer patients. Since the main clinical trials excluded immunocompromised patients including cancer patients, there was a concern of the applicability of the reported safety and efficacy of the vaccines in cancer patients. Additionally, concerns regarding the effect of cancer treatments on the vaccine’s safety and its possible interaction with cancer treatments were expressed. In one study that included 767 cancer patients, there were 447 unvaccinated individuals among them, of which 52% reported their preference to end cancer treatment before receiving the vaccine [3]. Patients from another study were most concerned that vaccine-related adverse events would worsen current anti-cancer therapy side effects (29%) and that there is not enough information regarding the safety of the COVID-19 vaccine in cancer patients undergoing oncological therapy (27%) [93].

### 4.2. Significantly Associated Factors with Vaccine Hesitancy/Acceptance

Several studies investigated factors that were associated with increased vaccine hesitancy. Mistrust in the health care system (OR 8.79, 95% CI [4.26–18.15]), noncompliance with prior influenza immunization (OR 2.27, 95% CI [1.57–3.29]), and low educational attainment (OR 1.84, 95% CI [1.17–2.89]) were all associated with increased vaccine hesitancy [90]. Higher education was also associated with increased vaccine acceptance (*p* = 0.0056) [89]. Interestingly, patients who routinely received the influenza vaccine were much more accepting of COVID vaccination, with 91.6% acceptance (14,905/16,269) compared to the 45.9% (2083/4545) acceptance prevalence among those who did not routinely receive an influenza vaccine (*p* < 0.001) [94]. In a separate study of 200 Tunisian cancer patients, the willingness to receive influenza vaccine was significantly associated with COVID-19 vaccine acceptance (OR = 3.9, 95% CI [1.6–9.3]; *p* = 0.002) [95]. These studies suggest that a general distrust in vaccines or a lack of appreciation of the severity of COVID-19 infections may contribute to the vaccine hesitancy expressed among cancer patients. A general distrust in the country’s healthcare system and its ability to effectively rollout a vaccination program has also been associated with vaccine hesitancy [96]. A study conducted in China that included 744 breast cancer survivors revealed that vaccine hesitancy or refusal was expressed by over 73% of the respondents [97]. The primary reason for hesitancy or refusal in 46% of patients was the lack of knowledge about the safety of the vaccines for cancer patients. Factors associated with vaccine hesitancy/refusal included current endocrine or targeted therapy (OR 1.52, 95% CI [1.03–2.24]) and no notification from communities or units (OR 2.46, 95% CI [1.69–3.59]). This demonstrated that many cancer patients were unaware of the effect of COVID-19 vaccines on cancer treatments and preferred to avoid the risk of any possible complications. Similarly, in a separate study, current endocrine or targeted therapy was associated with increased vaccine hesitancy (OR 1.52, 95% CI [1.03–2.24]) [97].

### 4.3. How to Combat Vaccination Hesitancy among Cancer Patients?

**Figure 2:**
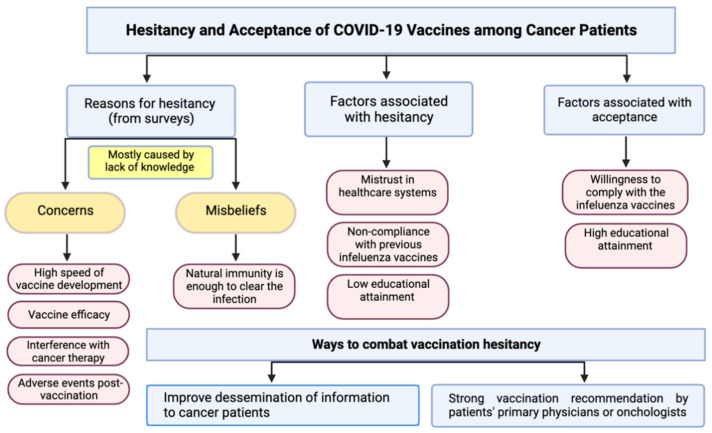
Reasons for and factors associated with vaccination hesitancy or acceptance among cancer patients compiled from studies which conducted surveys in different countries and the suggested ways to combat such hesitancy.

Current guidelines indicate that COVID-19 vaccines are safe and recommended in the majority of cancer patients, including patients undergoing therapy, with few exceptions. Better education and dissemination of information to patients with cancer is needed to combat vaccine hesitancy. Several ways to combat vaccine hesitancy have been suggested, the most prominent being the increased advocacy by oncologists and primary physicians. Patients indicated that they are most likely to listen to their oncologists regarding recommendations of vaccination. In a German survey of 425 cancer patients, around 85% of participants claimed to trust their attending physician’s recommendations regarding the COVID-19 vaccines [98]. Similarly, Villarreal-Garza et al., reported that 64.5% of the hesitant patients would consider receiving the vaccine if recommended by their oncologists [90]. Marijanović et al., conveyed that the majority of participants (82.4%) stated recommendation by their oncologist could influence their decision about vaccination [91]. One study in Korea showed that the initial rate of vaccine acceptance was 61% of the 1001 cancer patients surveyed. The rate increased to 91% of participants who received their attending physician’s recommendation for vaccination [99]. Results from several other studies further support the notion that recommendations coming from a patient’s oncologist would be well received by cancer patients [97,100]. Therefore, it is imperative that all oncological providers are well informed of the most accurate and up-to-date recommendations regarding vaccination and to start discussions with their patients to address their concerns. Figure 2 summarizes the reasons behind, and factors associated with vaccination hesitancy or acceptance among cancer patients compiled from the above-mentioned studies and the suggested ways to combat such hesitancy.

## 5. Ongoing Clinical Trials and Future Challenges

As of date, fifteen interventional/clinical trials that explore the immunogenicity and safety of COVID-19 vaccines in cancer patients are listed on ClinicalTrials.gov [101]. These studies, outlined with their identifiers in Table 1, take place worldwide across Europe, Asia, North America, and Australia. The listed trials include participants with both hematologic & solid malignancies; a few of the trials focus on immunocompromised patients, including transplant recipients, patients with human immunodeficiency virus (HIV), or patients with malignancy or autoimmune disease receiving chemotherapy, radiotherapy, anti-PD-1 therapy, anti-PD-L1 therapy, or anti-CD20 therapy. Three of the trials have published results which demonstrated lower seropositivity rates in cancer populations compared to healthy controls, but highlight the ability for cancer patients to achieve seroconversion and an increase in anti-SARS-CoV-2 antibody following a third booster dose of the vaccine [1,102,103]. As most of these trials are still ongoing, shortcomings have not yet been clearly identified, but the finalized trials indicated a need for studies with larger sample sizes to determine the effective vaccine type and dosage appropriate for cancer patients, with the aim to provide protection against COVID-19 without disrupting cancer therapy [1].

## 6. Discussion

**Figure 3:**
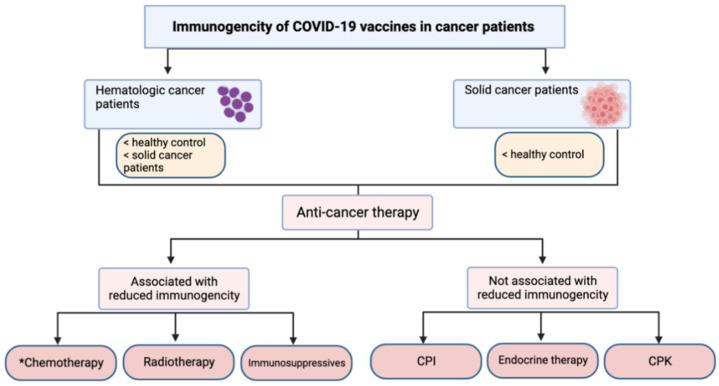
Immunogenicity of COVID-19 vaccines in cancer patients with hematologic or solid cancers and the effect of different types of anti-cancer therapy on the immunogenicity. Immunogenicity was measured in most of the studies by rates of seropositivity and/or antibody titers. * Only chemotherapy was reported to reduce both rates of seropositivity and antibody titers.

The initial clinical trials for COVID-19 vaccines did not provide data for cancer patients. Despite this, not many large-scale trials studying the efficacy of COVID-19 vaccines in cancer patients exist, though smaller trials around the world have provided some evidence. Cancer patients were found to have significantly diminished serological response after the first dose of the COVID-19 vaccine, but the second dose indicated an increased immune response but was still reduced when compared to healthy controls [17]. Antibody titers were lower in patients with hematological malignancies as compared to solid tumors [17]. Furthermore, chemotoxic treatments showed a diminishing effect on the serological antibodies [17,28]. Immunotherapy such anti-CD20 therapy and targeted therapies like tyrosine kinase inhibitors also resulted in a drop in serological response. Both chemotherapy and immunotherapy showed a positive correlation between time lapsed after treatment and serological response. Patients receiving only CPI were found to have higher titers of antibodies when compared to other combinations of treatments [52]. Data are still lacking on the difference of immunogenicity in cancer patient between different vaccines as most clinical trials did not assess or include cancer patients. For instance, cancer patients with immunodeficiency, as well as those receiving radiotherapy and chemotherapy were not allowed to be part of the Phase III clinical trial of Pfizer’s COVID-19 vaccine due to concerns about immunosuppression [104]. Overall, even though the immunological response is not as robust in cancer patients, most trials still report a long-term seropositivity of 70–88% [52]. Figure 3 summarizes the effect of different factors on the immunogenicity of COVID-19 vaccines in cancer patients including the different types of anti-cancer therapy.

Cancer treatments impair immunogenicity through different modes of actions. Cytotoxic chemotherapies interfere with DNA replication and synthesis and can disrupt the proliferation of lymphocytes during immune activation. Similarly, targeted therapies like anti-CD20 agents, tyrosine kinase inhibitors, and CAR T-cell therapy can severely deplete peripheral B-cell populations and affect various cytokine pathways needed to respond to vaccine introduced antigens [17]. An exception to this includes immune check-point inhibitors, a form of immunotherapy that enhances the immune system to detect and target cancerous cells. This could explain why cancer patients being treated with immunotherapy alone have a higher serological response than those on other combinations of treatments.

Several new trials have reported that COVID-19 vaccines have shown similar safety profiles in cancer patients as compared to the general population. The most common local and systemic side effects were pain at injection site, myalgia, and fatigue [17]. These were mostly mild to moderate in severity in both the general population and patients with cancer [17]. Notably, lymphadenopathy being an expected side effect of the Pfizer vaccine may alarm cancer patients as they may attribute swollen lymph nodes to malignancy [105]. No difference in adverse events was observed between hematological or solid malignancies or between patients undergoing cancer treatment and those who were treatment-naïve [4,17,103].

Immune-related adverse events, although rare, are still observed in the general population. However, in cancer patients these immune-related adverse events might be triggered by immune dysregulation caused by the underlying disease pathophysiology of malignancy as well as the effects of different treatments. One group of treatments that has posed a particular concern are CPI, which are being increasingly used to treat cancer patients [106]. These agents activate the immune system by targeting pathways that regulate programmed cell death (PD-1 and cytotoxic T-lymphocyte-associated protein 4 (CTLA-4) in T-cells [107]. This immune activation can, on the one hand, enhance the immunogenicity of COVID-19 vaccines and can, on the other hand, lead to uncontrolled immune activation, triggering an “inflammatory storm” in response to vaccine components [107]. No increased incidence of immune-related adverse events have been observed in cancer patients being treated with CPI [108]. However, vaccine administration to patients on combined CPI therapy (anti-PD-1, anti-PD-L1 and anti-CTLA-4) causes some concern due to the possible risk of immune-related adverse events [109]. It was suggested that myelitis may occur due to interleukin-6 (IL-6) and interleukin-17 (IL-17) running an inflammatory response leading to cytokine storm [110]. One example is atezolizumab, a monoclonal antibody which binds to tumor cells expressing PD-L1; this inhibits binding with T-cell expressed PD-1 and B7.1 receptors, allowing activation and proliferation of T-cells and enhanced function and memory cell formation to fight the tumor. Vaccine -induced causes of inflammation are less known, with active antigens in the vaccine or other constituents such as adjuvants causing inflammation. Some could also have an autoimmune reaction in the presence or absence of molecular mimicry [110]. Au et al., suggested that immune checkpoint therapy blocks PD-1, allowing for T-cell proliferation, causing patients to have an increased baseline of activated T-cells. When the vaccine was given, this tipped the immune system to CRS. However, S-reactive T-cells were not detected in the periphery, making this less likely. However, the T-cells associated with CRS could reside in tissue or lymph nodes, making them undetectable in blood [67]. Mei et al., speculated that giving the COVID-19 vaccine and anti-PD1 therapy within close temporal proximity of each other may enhance co-stimulatory and reduce co-inhibitory regulation, accounting for the increase in mild AEs in vaccinated patients. Additionally, they also speculated that this increase in immune response allows for the chemotherapy to work more effectively, consistent with their finding of vaccinated patients having higher disease control rates [66].

## 7. Conclusions and Recommendations

This review focused on the immunogenicity and safety of COVID-19 vaccination among cancer patients, as well as vaccine hesitancy. Malignancy was described to lower seropositivity rates, most notably in patients with hematologic malignancies compared to those with solid cancers. While active cancer therapy in general showed significantly lower seroconversion rates, specific cancer therapies associated with decreased vaccine immunogenicity included chemotherapy, radiotherapy, and immunosuppressives, as compared to healthy controls. Chemotherapy specifically showed lower seropositivity and antibody titers compared to cancer patients receiving other treatments. On the other hand, CPI, endocrine therapy, and CDK inhibitors were not found to affect seropositivity. Many effects of therapy on immunogenicity are not well described but are likely to be secondary to a medication mechanism of action.

COVID-19 vaccination amongst individuals with cancer is well-tolerated and generally safe, with similar rates of adverse events in patients without cancer, with a history of cancer, or those receiving active treatment compared to healthy controls. Though rare, radiation recall phenomenon was reported in those receiving radiotherapy, and higher rates of muscle pain was noted in patients receiving CPI. Post-vaccination lymphadenopathy was a common adverse event that may be mistaken for malignancy despite being of inflammatory origin. Despite the overall safety profile, vaccine hesitancy remains due to lack of knowledge regarding compatibility with disease, risks, efficacy, side effects, and concerns about interactions with cancer therapies. A lack of information regarding vaccine safety among this population, as well as a lack of appreciation for the severity of COVID-19 infections further contribute to hesitancy. Factors associated with increased hesitancy include mistrust in the healthcare system, non-compliance with prior influenza immunization, and low educational attainment; increased acceptance was seen among patients with higher education and those willing to routinely receive the influenza vaccine.

Despite the reported lower immunogenicity of the COVID-19 vaccines in cancer patients receiving certain types of anti-cancer treatments as compared to healthy controls or to cancer patients receiving other types of treatments, booster doses were noted to statistically increase seroconversion rates amongst various cancer types and treatments, and therefore were recommended for cancer patients. Furthermore, neither cancer nor cancer therapy were viewed as contraindications to COVID-19 vaccination in the reviewed studies. Regarding lymphadenopathy, it is recommended that vaccination is not delayed, and that vaccination history is provided to the radiologist if a patient is scanned for concerning lymphadenopathy. To reduce false positive nodal findings post-vaccination, the history of COVID-19 vaccinations, number of doses and dates, as well as site and side of injection should be strictly documented at time of vaccine administration. As it may be difficult to distinguish between benign and malignant hyperactivity in lymph nodes, it is recommended that patients with breast cancer, axillary lymphoma, and malignancy of the upper limb should not undergo vaccination in the arm that has a lymph node with expected nodal drainage of a tumor. Vaccine hesitancy can be combatted with better education and dissemination of information to cancer patients, and patients generally demonstrated more compliance when primary physicians and oncologists recommended the vaccine and provided sufficient information.

## Figures and Tables

**Table 1:** Current Clinical Trials Investigating COVID-19 Vaccination in Patients with Cancer or Immunocompromised States.

Study Title & ClinicalTrials.gov Identifier	Country	Study Phase	Primary Outcome Measures	Study Participants & Inclusion Criteria	Intervention & Model	Results
Evaluation of the Effect and Side Effect Profile of COVID-19 Vaccine in Cancer Patients Identifier: NCT04771559 [1,101]	Turkey	Complete	COVID-19 antibody titers. Time frame: 1 month	N = 1500 Patient group: Individuals aged 18+ diagnosed with cancer, who had received two doses of the COVID-19 vaccine Control group: Individuals aged 18+ with no history of cancer, who had received two doses of the COVID-19 vaccine	Intervention: COVID-19 antibody test. Non-randomized, parallel assignment	Seropositivity rates at one month: Patient group: 85.2% Control group: 97.5% (*p* < 0.001) Lower seropositivity in cancer patients associated with chemotherapy and age 60+ (*p* < 0.001)
Immune Response to the COVID-19 Vaccine Identifier: NCT04936997 [101,102]	USA	Early Phase 1	Immune response to 2nd COVID-19 vaccination booster (3rd vaccine) in patients with solid malignancies on immunosuppressive therapy Time frame: 3 months	N = 20 Patient group: Individuals aged 18+ with active solid tumor malignancy on active chemotherapy, who had received two doses of the Pfizer COVID-19 vaccine	Intervention: SARS-COV2 Pfizer Vaccine Single group assignment	80% (16/20) of participants demonstrated a median threefold increase in antibody response one week following a third dose of the Pfizer vaccine. No improvement was noted in T-cell responses. Adverse events were mild in nature.
Impact of the Immune System on Response to Anti-Coronavirus Disease 19 (COVID-19) Vaccine in Allogeneic Stem Cell Recipients (Covid Vaccin Allo) Identifier: NCT04951323 [101]	Belgium	Phase 3	Quantification of anti-SARS-CoV-2 IgG antibodies after vaccination in allogenic stem cell recipients Time frame: 49 days following first injection	Estimated N = 50 Patient group: Individuals aged 18+ who had undergone allogeneic hematopoietic stem cell transplantation 3 months to 5 months prior. Patients were excluded if they had active malignant disease at the time of inclusion	Intervention: Anti-COVID19 mRNA-based vaccine (BNT162b2, Comirnaty^®^, commercialized by Pfizer) Single group assignment	N/A
Safety and Immunogenicity of COVID-19 Vaccination in Patients With Cancer Identifier: NCT05018078 [101]	China	N/A	Primary Outcome 1: Safety of the COVID-19 vaccine, monitoring the occurrence of adverse effects Time frame: Within 2 months following the first vaccine dose Primary Outcome 2: Immunogenicity of the COVID-19 vaccine, measuring antibody titers against SARS-CoV-2 Time frame: Within 2 months following the first vaccine dose	Estimated N = 300 Patient group: Individuals aged 18+, with a cancer diagnosis including hepatocellular carcinoma, breast cancer, lung cancer, esophageal cancer, gastric cancer or colorectal cancer. Individuals must have local or systemic anti-cancer therapies according to the treatment guidelines previously or currently, in stable condition with an Eastern Cooperative Oncology Group (ECOG) score below 2. Additionally, patients must have normal or basically normal multi-organ function, without contraindications to vaccination	Intervention: Coronavirus vaccine Single group assignment	N/A
A Trial of the Safety and Immunogenicity of the COVID-19 Vaccine (mRNA-1273) in Participants With Hematologic Malignancies and Various Regimens of Immunosuppression, and in Participants With Solid Tumors on PD1/PDL1 Inhibitor Therapy, Including Booster Doses of Vaccine Identifier: NCT04847050 [101]	USA	Phase 2	Primary Outcome 1: Safety and reactogenicity of the mRNA-1273 vaccine, soliciting local and systemic adverse reactions 7 days after each injection, and unsolicited adverse events up to 28 days post-injection Time frame: 14 months Primary Outcome 2: Immunogenicity of the mRNA-1273 vaccine in patients with a hematological malignancy and are immunosuppressed due to their disease, and/or receiving PD-1/PDL-1 inhibitor for treatment of a solid tumor. Measured titers of specific binding antibody (bAb) on day 1, 29, 36, 57, 209, and 394 Time frame: 14 months	Estimated N = 220 Patient group: Individuals aged 18+ with either: - Solid tumor diagnosis, receiving PD1/PDL1 inhibitor treatment, - Diagnosis of acute leukemia (myeloid (AML) or lymphoid (ALL) or other); multiple myeloma; Waldenstrom macroglobulinemia, or - Diagnosis of lymphoma, including chronic lymphocytic leukemia Individuals must demonstrate adequate organ and bone marrow function on laboratory assessment within 4 weeks of vaccine administration	Intervention: mRNA-1273 injection. Non-randomized, parallel assignment	N/A
The Immune Reaction Upon COVID-19 Vaccination in the Belgian Cancer Population. Identifier: NCT05033158 [101]	Belgium	N/A	Immune response measuring quantification of anti-SARS-CoV-2 IgG antibodies (against full Spike, S1, S2, RBD, and N proteins) 4 weeks after first vaccine administration Time frame: 4 months	Estimated N = 3000 Patient group: Individuals aged 18+ with oncological or hematological malignancy, or a history of it, with a life expectancy >3 months	Intervention: Blood sampling Single group assignment	N/A
SARS-CoV-2 Vaccine (COH04S1) Versus Emergency Use Authorization SARS-COV-2 Vaccine for the Treatment of COVID-19 in Patients With Blood Cancer Identifier: NCT04977024 [101]	USA	Phase 2	Biological response, based on at least a 3-fold increase in anti-SARS-CoV-2 antibodies or interferon gamma levels Time frame: At 28 days post the second vaccine injection	Estimated N = 240 Patient group: Individuals aged 18+ with hematologic malignancy and an ECOG score of 2 or less. They must have received either allogenic or autologous hematopoietic cell transplant, or cellular therapy (chimeric antigen receptor [CAR] T-cell) therapy and be at least 3 months post treatment infusion	Interventions: COVID-19 Vaccine, Diagnostic Laboratory Biomarker Analysis, and Synthetic MVA-based SARS-CoV-2 Vaccine COH04S1 Randomized. parallel assignment	N/A
Safety and Immunogenicity of Prime-boost Vaccination of SARS-CoV-2 in Patients With Cancer Identifier: NCT05273541 [101]	China	Phase 1 Phase 2	Primary Outcome 1: Determining the safety of the prime-boost vaccine, measuring the occurrence of adverse effects post-vaccination Time frame: Within 1 week after the prime-boost vaccination Primary Outcome 2: Determining immunogenicity by titers of anti-SARS-CoV-2 antibodies Time frame: Within 3 months after the prime-boost vaccination	Estimated N = 100 Patient group: Individuals aged 18+, with a cancer diagnosis including hepatocellular carcinoma, breast cancer, lung cancer, esophageal cancer, gastric cancer or colorectal cancer. Individuals must have local or systemic anti-cancer therapies according to the treatment guidelines previously or currently, in stable condition with an ECOG score below 2. Additionally, patients must have normal or basically normal multi-organ function, without contraindications to vaccination.	Intervention: Coronavirus vaccination Single group assignment	N/A
Study Evaluating SARS-CoV-2 (COVID-19) Humoral Response After BNT162b2 Vaccine in Immunocompromised Adults Compared to Healthy Adults Identifier: NCT04952766 [101]	France	Phase 4	Protective humoral response post-vaccination, measuring the proportion of immunocompromised individuals with neutralizing activity against the “Wuhan” stain of SARS-CoV-2, as compared to healthy subjects Time frame: 2 months	N = 196 Adult volunteers belonging to one of the following groups: Immunocompromised group (~15 participants per subgroup): - Kidney transplant - Extracorporeal dialysis - Solid cancer, receiving chemotherapy and/or radiotherapy - Myeloma, receiving chemotherapy - Hematologic malignancy, receiving chemotherapy - Diseases treated with anti-CD20 (or, patients not treated at the time of vaccination, but will be immediately after) - Multiple sclerosis, receiving anti-CD20 (or, patients not treated at the time of vaccination, but will be immediately after) - Common variable immune deficiency, or other causes of severe hypogammaglobulinemia requiring chronic treatment with polyvalent immunoglobulin - Malignant tumor, receiving anti-PD1 or anti-PDL1 therapy - HIV - Complicated type 2 diabetes (with micro and/or macroangiopathy) Non-immunocompromised group: vaccinated with either Comirnaty TM or AstraZeneca’s Vaxzevria TM for the first dose	Intervention: Biological samples Single group assignment	N/A
COVID-19 VAX Booster Dosing in Patients With Hematologic Malignancies Identifier: NCT05028374 [101]	USA	Phase 2	Seroconversion rates of anti-SARS-CoV-2 antibody following a booster dose of the Moderna mRNA COVID-19 vaccine Time frame: 28 (±3 days) following booster dose	N = 119 Patient group: Individuals aged 18+ who have been previously diagnosed with multiple myeloma (MM)/amyloid light-chain amyloidosis, or other hematologic malignancy. They must have previously received any one of the available COVID-19 vaccines between 4–36 months prior to study enrollment, with anti-SARS-CoV-2 IgG titers less than 1.0 unit, or between 1.0–1.99 units. If patients are currently receiving potentially immunosuppressive cancer therapy, a two-week interruption before and after the booster dose of the vaccine is encouraged, but not required (at physician discretion)	Intervention: A single “booster” dose of the Moderna mRNA COVID-19 vaccine Single group assignment	N/A
Booster Dose Trial Identifier: NCT05016622 [101]	USA	Phase 2	Rates of seroconversion for SARS-CoV-2 anti-spike antibody Time frame: 4 weeks after booster dose	Estimated N = 100 Patient group: Individuals aged 18+ with a known diagnosis of any malignancy (either active or post completion of therapy), with negative SARS-CoV-2 spike IgG at least 14 days post-2nd dose of an mRNA-based COVID-19 vaccine, or 28 days after a single dose of the adenovirus-based Johnson & Johnson vaccine	Intervention: BNT162b2 vaccine Single group assignment	N/A
Passive Antibodies Against COVID-19 With EVUSHELD in Vaccine Non-responsive CLL Identifier: NCT05465876 [101]	Canada	Phase 2	Conferring passive immunity to CLL patients, measuring the proportion of participants with anti-spike antibodies after EVUSHELD administration Time frame: 12 months	Estimated N = 200 Patient group: Individuals aged 18+ with a diagnosis of CLL, who are either treatment-naïve, post-treatment, or on-treatment for CLL, and an ECOG score between 0–2. They must have received at least two doses of the Pfizer, Moderna, or AstraZeneca COVID-19 vaccines between 28 days-18 months prior to enrollment, demonstrating absent or suboptimal response. Participants must weigh at least 40 kg, have adequate organ function laboratory values, and have a life expectancy >6 months	Intervention: EVUSHELD Single group assignment	N/A
Bringing Optimised COVID-19 Vaccine Schedules To ImmunoCompromised Populations (BOOST-IC): an Adaptive Randomised Controlled Clinical Trial Identifier: NCT05556720 [101]	Australia	Phase 3	Measuring the geometric mean concentration (GMC) of anti-spike SARS-CoV-2 IgG antibody Time frame: 28 days after completion of vaccination trials	Estimated N = 960 Patient group: Individuals aged 16+ who have completed 3–5 doses of an Australian Therapeutic Goods Administration approved COVID-19 vaccine (Pfizer, Moderna, AstaZeneca, or Novavax). Patients must be in one of the following populations: - HIV infection, - Current recipient of a solid organ transplant, including kidney, pancreas, liver, malignancy episodes of severe rejection, requiring T- or B-cell depletion in the past 3 months, or - Undergoing chemotherapy, immunotherapy, and/or targeted therapy, or completed said therapies within the past 2 years in treatment of CLL, MM, or non-Hodgkin lymphoma	Interventions: BNT162b2, mRNA-1273, or NVX-COV2373 Randomized, parallel assignment	N/A
Anti-COVID-19 Vaccine in Children With Acute Leukemia and Their Siblings Identifier: NCT04969601 [101]	France	Phase 1 Phase 2	Primary Objective 1: Dose limiting toxicity, determined by the presence of grade ≥3 adverse events within 7 days following vaccine injection, that are deemed to be related to the vaccine Time frame: Within 7 days from first dose Primary Objective 2: Four-times or higher increase in the anti-spike IgG titer, AND positive anti-spike neutralizing test, indicating significant seroconversion Time frame: At 2 months from first dose	Estimated N = 150 Patient group: Individuals aged 1–15 years, with either: - Acute lymphoblastic leukemia (ALL), undergoing chemotherapy (within 2 weeks from the last injection) or for whom the last chemotherapy treatment was less than/equal to 12 months, or - Acute myeloid leukemia (AML) within 12 months from the end of treatment Control group: Healthy siblings aged 1–15 years, living in the same household as the child with ALL/AML more than 50% of the time	Intervention: Vaccine COMIRNATY^®^ (BNT162b2) Single group assignment	N/A
Safety, Efficacy of BNT162b2 mRNA Vaccine in CLL Identifier: NCT04862806 [101,103]	Israel	Complete	Primary Objective 1: Change in the number of participants with adverse events related to the BNT162b2 mRNA vaccine, assessed by a questionnaire with answers reported on a scale of 0–5 Time frame: 2–6 weeks after 2nd vaccination, 3 months after 2nd vaccination, 6 months after 2nd vaccination Primary Objective 2: Antibody persistence following the 3rd dose of the BNT162b2 mRNA vaccine in seronegative patients with chronic lymphocytic leukemia Time frame: 6 months	Estimated N = 1000 Patient group: Individuals aged 18+ with a diagnosis of CLL, who have received two 30-μg doses of BNT162b2 3 weeks apart	Intervention: COVID-19 serology Single group assignment	Of patients with CLL who failed to demonstrate a seropositive response following two doses of the BNT162b2 vaccine, nearly one fourth responded to a third dose of the vaccine. However, antibody responses were lower in patients undergoing active treatment, and patients with recent exposure (<12 months prior) to anti-CD20 therapy

Abbreviations: COVID-19, coronavirus disease 2019; USA, United States of America; SARS-CoV-2, severe acute respiratory syndrome coronavirus 2; IgG, immunoglobulin G; mRNA, messenger RNA; BNT162b2 and COMIRNATY, Pfizer BioNTech COVID-19 Vaccine; ECOG, Eastern Cooperative Oncology Group; mRNA-1273, Moderna COVID-19 Vaccine; PD-1, Programmed cell death protein 1; PD-L1, Programmed death-ligand 1; bAb, binding antibody; AML, acute myeloid leukemia; ALL, acute lymphoblastic leukemia; S1, spike protein subunit 1; S2, spike protein subunit 2; RBD, receptor-binding domain; COH04S1, City of Hope-developed COVID-19 vaccine; CAR T-cell, chimeric antigen receptor T-cell; MVA, modified vaccinia Ankara; CD-20, B-lymphocyte antigen CD20; HIV, human immunodeficiency virus; MM, multiple myeloma; EVUSHELD, tixagevimab and cilgavimab; CLL, chronic lymphocytic leukemia; GMC, geometric mean concentration; NVX-COV2373, Novavax COVID-19 Vaccine.
